# Association between plasma macrophage migration inhibitor factor and deep vein thrombosis in patients with spinal cord injuries

**DOI:** 10.18632/aging.101935

**Published:** 2019-04-29

**Authors:** Dong-Mei Wu, Zi-Hui Zheng, Shan Wang, Xin Wen, Xin-Rui Han, Yong-Jian Wang, Min Shen, Shao-Hua Fan, Zi-Feng Zhang, Qun Shan, Meng-Qiu Li, Bin Hu, Yuan-Lin Zheng, Gui-Quan Chen, Jun Lu

**Affiliations:** 1 Key Laboratory for Biotechnology on Medicinal Plants of Jiangsu Province, School of Life Science, Jiangsu Normal University, Xuzhou, P.R. China; 2 College of Health Sciences, Jiangsu Normal University, Xuzhou, P.R. China; 3 State Key Laboratory Cultivation Base for TCM Quality and Efficacy, School of Medicine and Life Science, Nanjing University of Chinese Medicine, Nanjing, P.R. China; 4 State Key Laboratory of Pharmaceutical Biotechnology, MOE Key Laboratory of Model Animal for Disease Study, Model Animal Research Center, Nanjing University, Nanjing, P.R. China; * Equal contribution

**Keywords:** macrophage migration inhibitory factor, deep vein thrombosis, spinal cord injuries, Chinese

## Abstract

The patients with spinal cord injury (SCI) suffered significantly higher risk of deep vein thrombosis (DVT) than normal population. The aim was to assess the clinical significance of macrophage migration inhibitory factor (MIF) as the risk factor for DVT in acute SCI patients. 207 Chinese patients were enrolled in this study, including thirty-nine (39) patients (18.8 %; 95 %CI: 13.5 %–24.2 %) diagnosed as DVT at the follow-up of 1 month. Nine (9) of the 39 patients (23.1%) were suspected of thrombosis before the screening. The MIF levels in plasma of DVT patients were significantly higher than DVT-free patients. The risks of DVT would be increased by 11 % (OR _unadjusted_: 1.11; 95% CI, 1.06–1.17, P<0.001) and 8 % (OR _adjusted_: 1.08; 1.03–1.14, P=0.001), for each additional 1 ng/ml of MIF level. Furthermore, after MIF was combined with established risk factors, area under the receiver operating characteristic curve (standard error) was increased from 0.82(0.035) to 0.85(0.030). The results showed the potential association between the high MIF levels in plasma and elevated DVT risk in SCI patients, which may assist on early intervention.

## INTRODUCTION

Spinal cord injury (SCI) has been one of the most fatal accidents, with an annual incidence rate of about 10.4 to 83 cases per million worldwide [1–2]. In China, previous study reported that the annual incidence rate ranged from 23.7 to 60.6 per million [3–4], thus nearly 800 00 peoples would be suffered from SCI every year.

Mechanical damage to the spinal cord leads to impairment to neurons, axons and glial lesions at the site of impact [5]. In the patients suffered from SCI, the process of neuronal damage would last for days to weeks, with primary acute neuronal injury and secondary neuronal loss due to the apoptosis or necrosis [6–7]. During this process, the timely and effective interventions can be performed to minimize the damage to axons and the support structures [1]. However, the opportunities for activity and full restitution remain limited [8].

Deep vein thrombosis (DVT) is a common and fatal complication during the first 2 weeks after SCI, accompanied with increased incidence and mortality [9–10]. A previous study has demonstrated that, the risk of DVT in SCI patients would be significantly raised, with an adjusted hazard ratio (HR) of 2.46-fold [11]. Miranda et al. [12] reported that DVT was related to motor nonfunctional paralysis. However, no effective intervention or prevention has been reported [12]. Therefore, the research and development of DVT accompanied with SCI would be urgently required.

The progressive neurodegeneration was resulted from persistent neuroinflammation after SCI [13]. The role of inflammation in pathogenesis of DVT had been proposed [14–15]. Macrophage migration inhibitory factor (MIF) has been suggested as a regulator of innate immunity. The functions of MIF in different acute and chronic inflammatory conditions as well as vascular pathology have been suggested [16–17]. Interestingly, one study found that the level of plasma MIF was increased in chronic SCI [18]. To date, no study has been performed on the associations between blood levels of MIF and risk of DVT in SCI patients. Our study was aimed to explore whether higher MIF levels could be a biomarker for predicting the increased risk of DVT in acute SCI patients.

## RESULTS

### Basic characteristics of patients

Finally, 207 SCI patients were included, including 74.9% (155/207) males, with the median age of 53(IQR, 37–62) years. In this study, a history of vein thrombosis was found in 45 patients (21.7%).

The ASIA scores were assessed at admission, including 51 cases of grade A, 41 cases of grade B, 38 cases of grade C, and 77 cases of grade D. 121 (58.5%) and 133(64.3%) patients suffered from cervical cord injuries and spinal fractures, respectively. The most common etiology of SCI was traffic accidents (46.4%), which followed as falls (31.4%). The median time was 16.0 (IQR, 9.0–21.5) hours from injury onset to blood collection. Before the diagnosis of DVT, no patient was treated with low-molecular-weight heparin as prophylactic antithrombotic therapy. Basic characteristics of SCI patients with or without DVT were listed (Table 1).

**Table 1:** Basal characteristic of SCI patients with DVT and without DVT^†^.

**Baseline characteristics**	**SCI patients**		***P*^‡^**
	**With DVT**	**Without DVT**	
N	39	168	*—*
Median age (yr, IQR)	59 (52–66)	48 (34–60)	0.010
Male sex, *n* (%)	31 (79.5)	124 (73.8)	0.48
Cigarette smoking, *n* (%)	21 (53.8)	76 (45.2)	0.33
Hypertension, *n* (%)	15 (38.5)	64(38.1)	0.97
Diabetes, *n* (%)	11 (28.2)	38(22.6)	0.46
Coronary heart disease, *n* (%)	14(35.9)	42(25.0)	0.17
History of VT, *n* (%)	14 (35.9)	31(18.5)	0.017
Time from onset to blood collected (hr, IQR)	16.5 (9.5–23.0)	16.0(8.5–21.0)	0.76
Etiologies, *n* (%)			0.86
Traffic accidents	18(46.1)	78(46.4)	
Falls	14(35.9)	51(30.6)	
Sports and violence	3(7.7)	19(11.3)	
Others	4(10.3)	20(11.9)	
Injury levels, n (%)			
Cervical injury	24(61.5)	97(57.7)	0.66
Thoracic injury	9(23.1)	24(14.3)	0.18
Lumbar injury	10(25.6)	25(14.9)	0.15
Combined fractures, n (%)			
Spinal fractures	29(74.4)	104(61.9)	0.14
Brain injuries	8(20.5)	37(22.0)	0.84
Other injuries	7(17.9)	17(10.1)	0.17
Clinical complications, n (%)	18(46.2)	41(24.4)	0.007
ASIA score, *n* (%)			0.24
A	14 (35.9)	37(22.0)	
B,	8(20.5)	33(29.6)	
C	7(17.9)	31(18.5)	
D	10(25.6)	67(39.9)	
Treatment, n (%)			
Surgery	10(25.6)	58(34.5)	0.29
Rehabilitation therapy	8(20.5)	67(39.9)	0.023
Hyperbaric oxygen therapy	8(20.5)	30(17.9)	0.70
Laboratory findings (Median, IQR)			
Glucose level, mmol/L	5.59 (5.13–6.32)	5.43 (4.92–6.30)	0.25
CRP, mg/L	7.7 (4.3–13.2)	4.5 (2.8–9.8)	0.009
IL-6, pg/ml	9.4(8.3–10.2)	8.3(7.0–9.6)	0.002
D-dimer, μg/L	320(215–395)	270 (170–324)	<0.001
MIF, ng/mL	27.2(22.3–32.5)	21.1(16.8–25.8)	<0.001

^†^Results are expressed as percentages or as medians (IQR)

^‡^Mann–Whitney U test or Chi-square test was used.

DVT: Deep vein thrombosis; SCI: spinal cord injuries; ASIA: The American Spinal Injury Association Impairment Scale; VT, vein thrombosis; CRP: C-reactive protein; MIF, Macrophage migration inhibitory factor; IL-6, Interleukin 6

### Main results

Thirty-nine patients (18.8 %; 95% CI: 13.5 %–24.2 %) were defined as DVT at the follow-up of 1 month. Before the screen, 9 of the 39 patients (23.1 %) were suspected of thrombosis. As showed in the Table 2, the age of patients with DVT were older, who suffered from higher frequencies of vein thrombosis higher, higher initial SCI severity and higher plasma D-dimer and CRP. In addition, patients with DVT had higher levels of IL-6.

**Table 2:** Univariate and multivariate logistic regression analysis for DVT.

**Predictor**	**Univariate analysis**			**Multivariate analysis^†^**		
	**OR^‡^**	**95% CI**	***P***	**OR^‡^**	**95% CI**	***P***
Age	1.03	1.01–1.05	0.010	1.02	1.01–1.05	0.040
History of vein thrombosis (Yes vs. no)	2.48	1.16–5.30	0.017	1.61	1.11–3.76	0.059
Clinical complications (Yes vs. no)	2.67	1.29–5.46	0.007	1.83	1.21–3.49	0.027
Rehabilitation therapy (Yes vs. no)	0.39	0.17–0.75	0.023	0.64	0.43–0.96	0.046
CRP	1.09	1.02–1.16	0.009	1.06	1.01–1.20	0.024
IL-6	1.41	1.14–1.74	0.002	1.21	1.05–1.63	0.013
D-dimer	1.01	1.00–1.02	<0.001	1.01	1.00–1.03	0.002
MIF	1.11	1.06–1.17	< 0.001	1.08	1.03–1.14	0.001

^‡^Note that the odds ratio corresponds to a unit increase in the explanatory variable.

^†^Adjusted for those significant risk factors which confirmed in the univariate analysis, including age, history of vein thrombosis, clinical complications, rehabilitation therapy, plasma levels of CRP, IL-6, D-dimer and MIF

OR, odds ratio; CI, confidence interval; CRP, C-reactive protein; DVT: Deep vein thrombosis; MIF, Macrophage migration inhibitory factor; IL-6, Interleukin 6.

**Figure 1:**
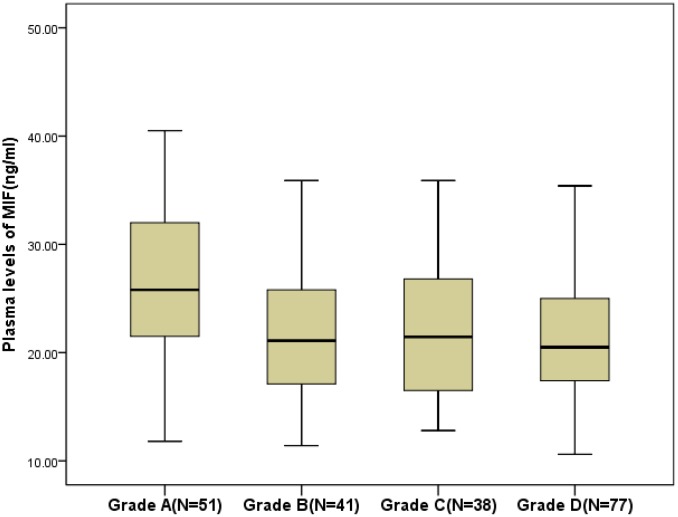
**Plasma levels of MIF in different groups divided according to the American Spinal Injury Association impairment scale (ASIA; Grade A to Grade D).** All data are medians and in-terquartile ranges (IQR). MIF=Macrophage migration inhibitory factor.

The MIF level in SCI patients was higher compared to that of in controls (22.1; IQR [17.4–27.8] ng/ml *vs.*15.0[11.2–20.1] ng/ml; P<0.001). The MIF levels were related with CRP (r=0.144, P=0.038) and IL-6 (r=0.228, P=0.001), while no relationship between MIF levels and sex, or age (P>0.05) was observed. A negative association between plasma MIF levels and SCI severity based on the ASIA score (r =−0.193, P=0.009) was showed. As shown in the Figure 1, MIF levels in plasma of patients with grade A (25.8; IQR, 21.4–32.2 ng/ml) were higher (P=0.002) than in grade B, (21.1; 17.1–26.5 ng/ml; P=0.017), grade C (20.5; 17.4–25.2 ng/ml; P=0.034) and grade D (21.5; 16.5–27.1 ng/ml).

**Figure 2:**
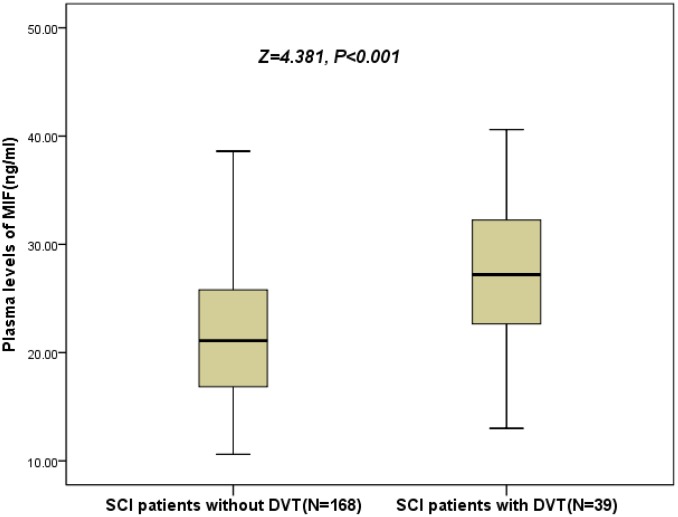
**Plasma levels of MIF in SCI patients with DVT and without DVT.** All data are medians and in-terquartile ranges (IQR); P values refer to Mann-Whitney U tests for differences between groups. MIF=Macrophage migration inhibitory factor; SCI= Spinal cord injuries; DVT= Deep vein thrombosis.

**Figure 3:**
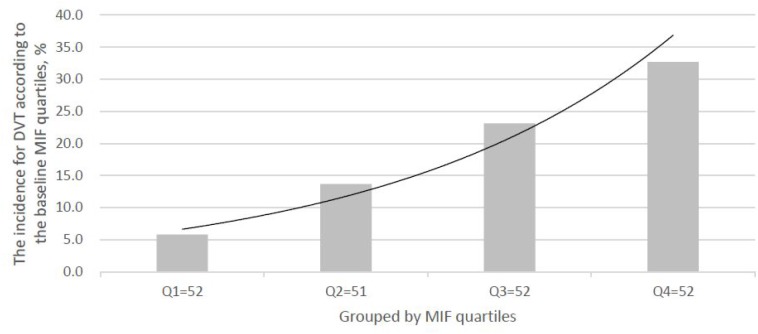
**The incidence for DVT according to the baseline MIF quartiles.** MIF in Quartile 1 (<17.4ng/ml), Quartile 2 (17.4–22.1ng/ml), Quartile 3 (22.2–27.8ng/ml), and Quartile 4 (>27.8ng/ml). MIF=Macrophage migration inhibitory factor; DVT= Deep vein thrombosis.

As shown in the Figure 2, the levels of MIF were also higher (P<0.001) in 39 patients developed DVT, than those of in DVT-free patients (27.2[22.3–32.5] ng/ml vs. 21.1[16.8–25.8] ng/ml; Z=4.381). As shown in the Figure 3, the risk of DVT was gradually elevated with increased quartiles of MIF. The risk of DVT according to MIF quartiles was ranged from 5.8 % (first quartile, Q1) to 32.7 % (fourth quartile, Q4) (P < 0.001).

ORs of MIF was calculated as risk indicators to predict DVT in univariate logistic regression analysis, and then it was compared with the established risk factors. For each additional 1 ng/ml of MIF, the risk of DVT was increased by 11% (OR _unadjusted_: 1.11; 95% CI, 1.06–1.17, P<0.001) and 8% (OR _adjusted_: 1.08; 1.03–1.14, P=0.001), respectively. There were also other significant DVT predictors, including age, vein thrombosis history, clinical complications, rehabilitation therapy, plasma CRP, D-dimer and IL-6. Multivariate analysis was applied to further explore the association between MIF and DVT. We estimated the OR _adjusted_ (95% CIs) of DVT according to quartiles of MIF (lowest quartile as the reference). As shown in the Table 3, in the multivariate models comparing Q2-Q4 against the Q1 of MIF levels, MIF in Q3 and Q4 was related with later developed DVT, and the risk of DVT was increased by 210% (OR _adjusted_: 3.10[1.05–9.33]; P=0.043) and 376% (4.76[2.55–11.75]; P<0.001), respectively. The multivariate model was applied to analyze the prognostic significance of combined modes including MIF and other significant clinical variables. The combined modes showed predictive value for DVT (OR for Q4, 2.15 [95% CI, 1.05–3.36; P=0.015]).

**Table 3:** Multivariate logistic regression analysis for DVT according to MIF Quartiles.

**MIF ^ǂ^**	**DVT/N**	**Crude OR (95%CI), P**	Multivariable-adjusted^‡*^, P
Quartile 1	3/52	Reference	Reference
Quartile 2	7/51	2.60(0.63–10.67), 0.018	—
Quartile 3	12/52	4.90(1.29–18.57), 0.012	3.10(1.05–9.33), 0.043
Quartile 4	17/52	7.93(2.16–29.16), <0.001	4.76(2.55–11.75), <0.001
Elevated vs. normal	17/52 *vs.* 22/155	2.94(1.41–6.12), 0.003	2.15(1.05–3.36), 0.015

^ǂ^ MIF in Quartile 1 (<17.4ng/ml), Quartile 2 (17.4–22.1ng/ml), Quartile 3 (22.2–27.8ng/ml), and Quartile 4 (>27.8ng/ml). Elevated MIF level was defined as greater than or equal to the 3^rd^ quartile level (≥27.8ng/ml).

^‡^Adjusted for those significant risk factors which confirmed in the univariate analysis, including age, history of vein thrombosis, clinical complications, rehabilitation therapy, plasma levels of CRP, IL-6, D-dimer and MIF

^*^P value for the trend <0.001

OR, odds ratio; CI, confidence interval; CRP, C-reactive protein; IL-6; Interleukin 6; MIF, Macrophage migration inhibitory factor; DVT: Deep vein thrombosis

The prediction of DVT by MIF was performed with ROC curves, with an AUC of 0.73 (95% CI: 0.64–0.81). The prognostic accuracy of MIF levels was higher than that of CRP (AUC: 0.66 [0.57–0.75]; P=0.001), age (0.63 [0.56–0.73]; P<0.001] and IL-6 (0.66 [0.57–0.76]; P=0.002), while was similar with D-dimer (0.75[0.67–0.83]; P=0.53), Table 4. The AUC can be improved in MIF and D-dimer combined model I (AUC: 0.80 (0.73–0.85); P=0.009). This improvement was stable in an internal 5-fold cross validation, the average AUC (± standard error) was 0.80 (±0.038) for the D-dimer and 0.75 (±0.042) for the combined model I, with a difference of 0.05 (±0.004). Furthermore, as showed in the Table 4, AUROC (± standard error) in combined models containing MIF and established risk factors was increased from 0.82 (± 0.035) to 0.85 (± 0.030), corresponding to combined model II vs. combined model III, with a significant difference of 0.03 (± 0.005) (P=0.028).

**Table 4:** Prediction of DVT according to ROC.

**Parameter**	**AUC**	**95% CI**		***p***
Prediction of DVT				
MIF	0.73	0.64	0.81	—
Age	0.63	0.56	0.73	<0.001
CRP	0.66	0.57	0.75	0.001
IL-6	0.66	0.57	0.76	0.002
D-dimer	0.75	0.67	0.83	0.53
Combined score I^†^	0.80	0.73	0.85	0.009
Combined score II^‡^	0.82	0.75	0.88	<0.001
Combined score III^††^	0.85	0.80	0.92	0.028

^†^including MIF and D-dimer. P value compared with D-dimer

^‡^including age, history of vein thrombosis, clinical complications, rehabilitation therapy, plasma levels of CRP, IL-6 and D-dimer

^††^including age, history of vein thrombosis, clinical complications, rehabilitation therapy, plasma levels of CRP, IL-6, D-dimer and MIF.

P value compared with combined score II.

AUC, area under the curve; CI, confidence interval; CRP, C-reactive protein.

## DISCUSSION

People with SCI would always suffer from severe physical disabilities. Secondary to the direct impairment to spinal cord, patients with SCI would develop various complications, including multiple organ dysfunction or failure [19]. MIF has been an important cytokine, which activate in innate immunity and inflammatory responses. It was relevant with pathophysiology of SCI [20–21] and vascular disease [22]. This study had investigated the association between MIF plasma levels and DVT risk in SCI patients. The research findings were as follows: (1) plasma MIF levels were elevated in SCI patients compared to that of in normal controls; (2) MIF levels in plasma were related to SCI severity based on ASIA; (3) higher plasma MIF were related to elevated risk of DVT in the follow-up of 1 month, for each additional 1ng/mL of MIF, the DVT risk before and after adjustment was increased by 11% and 8%, respectively; (4) MIF plasma levels at admission may be significant in predicting DVT of SCI patients.

Further, the reported incidence rate of DVT varied, ranged from 10% to 30% [23]. A previous study in Chinese patients with DVT found that 20.2% patients experienced DVT in the following 3-month after SCI [24]. Consistent with those results, our study showed that 18.8% of the SCI patients were determined as DVT in the follow-up of 1-month. Similarly, another study reported that 19.7% (55/279) of patients were diagnosed as DVT in the follow-up of 15 days [10]. However, a hospital-based retrospective epidemiological study (N=1340) from Guangdong, China showed that only 3.6% of the SCI patients suffered from DVT [25]. That mean that about 80% of the DVT patients were not diagnosed. Similarly, in this study, only 23.1% of the patients were suspected of thrombosis before the screened. Therefore, it's very meaningful for us to routinely screen for DVT in patients with SCI.

The low-grade chronic inflammatory state following SCI has been common, which was characterized by upgraded circulating proinflammatory mediators [26–27]. The significantly elevated levels of MIF were observed in SCI patients, both in our study and others [21]. The hypothalamic pituitary-adrenal axis was activated in SCI, thus improving MIF production by the pituitary gland [28]. Furthermore, highest levels of circulating inflammatory mediators were reported in acute SCI patients classified as ASIA grade A [29]. Similarly, we also showed that MIF levels were highest in acute SCI patients classified as ASIA grade A. Another study reported that high inflammatory indicators were positively associated with injury severity, which could be applied as outcome predictors [21].

The mechanism for the effects of MIF on DVT has not been clarified. The possible mechanism might be involved in inflammatory reaction. Acute inflammation might activate in thrombosis, and vascular biology [30]. The circulation of immune cells and pro-inflammatory mediators may be enhanced by SCI, thus activating the systemic inflammatory responses [19]. MIF is a multi-function cytokine participated in various immune and inflammatory processes. MIF, a pro-inflammatory cytokine, could bring out numerous pro-inflammatory molecules [31]. In a study performed on rat with traumatic spinal cord, the release of inflammatory mediators from astrocytes would be activated by MIF [32]. High levels of MIF would be endocytosed and then bind to Jab1, thus regulating cell growth and inflammation by binding to c-Jun and AP-1 [33]. ERK/MAPK would be transiently activated by MIF/Jab1 complexes in Src-dependent manner [34], which may be due to the noxious peripheral stimuli [35].

There were some limitations should be considered. First, information on the patients was limited, especially the factors affecting the MIF levels were not available, such as the infections or other complications. Further, the blood samples were collected at different time period. Second, the MIF level was only measured at admission after the SCI, while the pre-injury conditions could not be accurately reflected. In addition, the MIF was detected in plasma instead of cerebral spinal fluid (CSF), which may be not consistent with the conditions in the central nervous system. Third, patients who died in the follow-up were excluded. Those patients might also suffer from DVT, which lead to an underestimate. Fourth, the causal relationship could not be concluded with such observational study design. Interestingly, a previous study suggested the potential MIF inhibition in SCI [20]. Lastly, the subject number was limited in this study (N=207), which was come from one hospital. Thus, the conclusion in this study may not be generalized. In addition, this study was lack of parallel assessment of hypothalamic–pituitary–adrenal (HPA) axis functions and there was no information on MIF gene expression. The inflammatory diseases could be influenced by the polymorphisms in the promoter region of the MIF gene [36].

## METHODS

### Study population

During January 2015 and September 2018, the patients hospitalized with acute SCI in Emergency Department of Affiliated Hospital of Nanjing University of Chinese Medicine were enrolled. The SCI was identified with both clinical and radiographic results. The exclusion criteria: (1) malignant tumor; (2) previously diagnosed with vein thrombosis or other coagulation disorder; (3) liver or renal insufficiency; (4) any contraindications on heparin; (5) no informed consent, incomplete medical records or uncertain diagnosis and survival period less than 1 months; (6) less than 18 years; (7) non-acute SCI, traumatic brain injury (TBI) and severe abdominal trauma.

In addition, 100 age-and gender-matched volunteers without SCI were included as healthy controls. The study protocol was ratified by the ethics committee of Nanjing University of Chinese Medicine and performed per the rules of Helsinki Declaration. All included subjects signed the written informed consent before their participations in this study.

### Patient characteristics and DVT assessment

The basic information of patients was collected, including the age and sex, smoking history, and underlying diseases (hypertension, type 2 diabetes mellitus, cardiovascular disease and history of vein thrombosis). Furthermore, time of injury, cause of injury (traffic accidents, falls, sports and violence, and others), level of injury (cervical injury, thoracic injury and lumbar injury), severity of injury, combined fractures (spinal fractures, brain injuries and others), surgical history, hyperbaric oxygen therapy, rehabilitation therapy, clinical complications during the follow-up (pulmonary infection, urinary tract infection, bedsore, electrolyte disturbance, digestive system disease and others), and so on. At admission, SCI severity was evaluated with American Spinal Injury Association impairment scale (ASIA; A-D) score [37], which was assessed individually by two trained personnel through a structured interview with patients or closest relatives.

The DVT assessment was performed with color doppler ultrasonography (CDUS) on the SCI patients, regardless of existing symptoms. The real time B-mode ultrasonography was used to checked common femoral vein and the popliteal vein of patients. CDUS was used at 15, 21 and 30 days after injury, as well as whenever clinically requested. For patients show symptoms after checking, lower limb mechanical compression would be applied to prevent DVT, with external sequential pneumatic compression or elastic stockings [10].

### Clinical indicators

Fasting blood of all the patients was collected on the morning of the first day after the admission. The plasma was obtained by centrifuge at 1000 × g for 10 min and stored at -80°C. Plasma levels of MIF were tested by enzyme-linked immunosorbent assay kit (Catalog no. DMF00B; R&D Systems, Inc. Minneapolis, USA), with a measurement range of 2 ng/ml–100 ng/ml. The coefficients of variation (CV) for indicating the intra-assay and inter-assay were 6.0 %, 9.0 % and 4.5 %, 8.0 % at 1 ng/ml and 10ng/ml, respectively. Furthermore, the levels of plasma glucose, C-reactive protein (CRP), D-dimer, interleukin 6 (IL-6) were also detected with conventional established laboratory tests.

### Statistics

The discrete variable was expressed in the percentage or frequency, continuous variable was expressed in the medians (interquartile range, IQR). The comparison between groups was performed with chi-squared test or Mann–Whitney U-test. Bivariate correlation was performed with Spearman's Rank correlation. The effects of plasma MIF level on DVT were performed with binary logistic regression analysis (univariate and multivariate). The confounding factors were assessed and adjusted, expressed as adjusted odds ratio (OR) with 95% confidence interval (CI). The effects of elevated MIF levels on DVT risk was further estimated by MIF quartiles (lowest MIF quartile as the reference). The MIF level ≥ third quartile (Q3) was considered as elevated MIF. The receiver operating characteristic curve (ROC) was used and the area under ROC (AUROC) was performed for determining the cut-off points [38]. The logistic regression was used to evaluate the model of MIF combined with or without established risk factor (D-dimer) for predicting DVT risk. Lastly, difference between the model containing D-dimer (combined model II) and the model containing both D-dimer and MIF (combined model III) was also tested. All statistical analysis was performed with SPSS (version 22.0), with P < 0.05 defined as statistical significance.

## CONCLUSIONS

In summary, this study proposed that, for patients with SCI, the high MIF levels in plasma were associated with increased risk of DVT in the 1-month follow-up. Thus, MIF may be significant in identifying the risk of DVT in SCI patients and providing timely intervention. Further studies should be performed to clarify the mechanism between MIF levels and DVT risk, which may assist on proposing a new therapeutic strategy.
